# Detection of CLCF1 protein expression by flow cytometry

**DOI:** 10.1038/s41598-024-64101-9

**Published:** 2024-06-10

**Authors:** Véronique Laplante, Marine Rousseau, Félix Lombard-Vadnais, Ulysse Nadeau, Agathe Nazha, Jean-François Schmouth, Mukut Sharma, Sylvie Lesage, Jean-François Gauchat, Sarah Pasquin

**Affiliations:** 1 Département de pharmacologie et physiologie, Université de Montréal, Montréal, QC H3T 1J4 Canada; 2 Département de microbiologie, infectiologie et immunologie, Université de Montréal, Montréal, QC H3T 1J4 Canada; 3 Centre de recherche de l’Hôpital Maisonneuve-Rosemont, Université de Montréal, Montréal, QC H1T 4B3 Canada; 4 Centre de recherche du CHUM, Université de Montréal, Montréal, QC H2X 0A9 Canada; 5 Renal Division, Kansas City Veterans Affairs Medical Center, Kansas City, MO 64128-2226 USA

**Keywords:** Cytokines, T cells

## Abstract

Cardiotrophin-like cytokine factor 1 (CLCF1) is an IL-6 family cytokine with neurotrophic and immuno-modulating functions. CLCF1 mRNA has been detected in primary and secondary lymphoid organs, and up-regulation of CLCF1 mRNA levels has been associated with the T helper (Th) 17 polarization. However, information regarding CLCF1 expression by immune cells at the protein level remains scarce. We have developed a methodology that uses a monoclonal antibody (mAb) directed against CLCF1 for the detection of human and mouse CLCF1 by flow cytometry. We have successfully detected CLCF1 protein expression in cells from the mouse pro-B cell line Ba/F3 that were transduced with CLCF1 cDNA. Interestingly, we found that the anti-CLCF1 mAb inhibits CLCF1 biological activity in vitro by binding to an epitope that encompasses site III of the cytokine. Moreover, we have detected CLCF1 expression in mouse splenic T cells, as well as in vitro differentiated Th1 cells. The specificity of the fluorescence signal was demonstrated using *Clcf1*-deficient lymphocytes generated using a conditional knock-out mouse model. The detection of CLCF1 protein by flow cytometry will be a valuable tool to study CLCF1 expression during normal and pathological immune responses.

## Introduction

Cardiotrophin-like cytokine factor 1 (CLCF1) is a cytokine of the IL-6 family that was initially known as novel neurotrophin-1, B cell stimulating factor 3 and cardiotrophin-like cytokine^1–3^. CLCF1 activates the tripartite ciliary neurotrophic factor receptor (CNTFR), which is composed of CNTFRα, leukemia inhibitory factor receptor β (LIFRβ) and gp130^4^. Similar to other receptors that use the gp130 chain, the CLCF1-CNTFR axis signals through the JAK/STAT3 pathway^5^. Although CLCF1 contains a putative signal peptide, it is not secreted when expressed alone, and is believed to be trapped in the cell secretory pathway^4^. To be secreted, CLCF1 needs to form a heterodimeric complex with the cytokine receptor-like factor 1 (CRLF1)^4^ or the soluble form of CNTFRα^6^. CLCF1 is highly conserved across vertebrate species, with 97% identity between human and mouse CLCF1^7^, which suggests important and non-redundant functions for this cytokine.

CLCF1 exhibits immuno-modulating activities on B cells and myeloid cells. Specifically, injections or overexpression of CLCF1 in mice leads to B cell hyperplasia in secondary lymphoid organs, as well as IgG and IgM hyperglobulinemia^1,2^. Administration of CLCF1 to mice also results in an increased number of circulating CD11b^+^ myeloid cells^8^. A recent study has implicated CLCF1 in the regulation of thermogenesis, and showed that elevated levels of CLCF1 suppress mitochondrial biogenesis leading to the whitening of brown adipose tissue^9^. Finally, CLCF1-CRLF1 administration has been shown to decrease pulmonary fibrosis and lead to the pulmonary accumulation of CD4^+^ T cells in a mouse model of idiopathic pulmonary fibrosis^10^.

CLCF1 mRNA can be detected in various organs, such as the lymph nodes, spleen, bone marrow, lungs, ovaries and uterus^2,3^, as well as in diverse cell types, such as A2 astrocytes^11^ and megakaryocytes^12^. The group of Dr. Sweet-Cordero has also detected the presence of both CLCF1 and CRLF1 mRNA in mouse and human cancer-associated fibroblasts. Their study indicates that CLCF1-CRLF1 can act as a secretory factor to stimulate tumor growth in non-small cell lung cancer^13^. Finally, Cleret-Buhot et al*.* have shown in healthy donors that CLCF1 transcripts are upregulated in CCR4^+^ CCR6^+^ Th17 cells compared to CXCR3^+^ CCR6^-^ Th1 cells^14^.

Although CLCF1 mRNA has been detected in many organs and cell types, information on CLCF1 protein expression remains scarce. With increasing evidence that CLCF1 plays critical roles in many pathologies such as cancer and pulmonary fibrosis, it is vital to elucidate CLCF1 protein expression pattern in primary cells to further our understanding of CLCF1 function and how to best target it in a therapeutic setting. Flow cytometry on fixed and permeabilized cells is extensively used to investigate normal and pathological cytokine expression by immune cells, as well as to discriminate the function of immune cells based on their cytokine synthesis patterns^15^. Therefore, we have optimized a flow cytometry protocol for the detection of CLCF1, and showed that it can be used to assess the expression of CLCF1 in primary mouse immune cells and in vitro differentiated T cells. This current procedure differs from our previous attempt to detect CLCF1^16^ in that (i) it can be performed on both the mouse and human cytokine and (ii) it relies on a commercial mAb. To confirm the specificity of the flow cytometry fluorescence signal, we used a *Clcf1* conditional knock-out mouse model. Our investigation also showed that the anti-CLCF1 mAb neutralizes CLCF1 activity in vitro, indicating that it can be used to study the function and pathological roles of CLCF1 in preclinical models.

## Results

### CLCF1 can be detected by flow cytometry in transduced Ba/F3 cells

**Figure 1:**
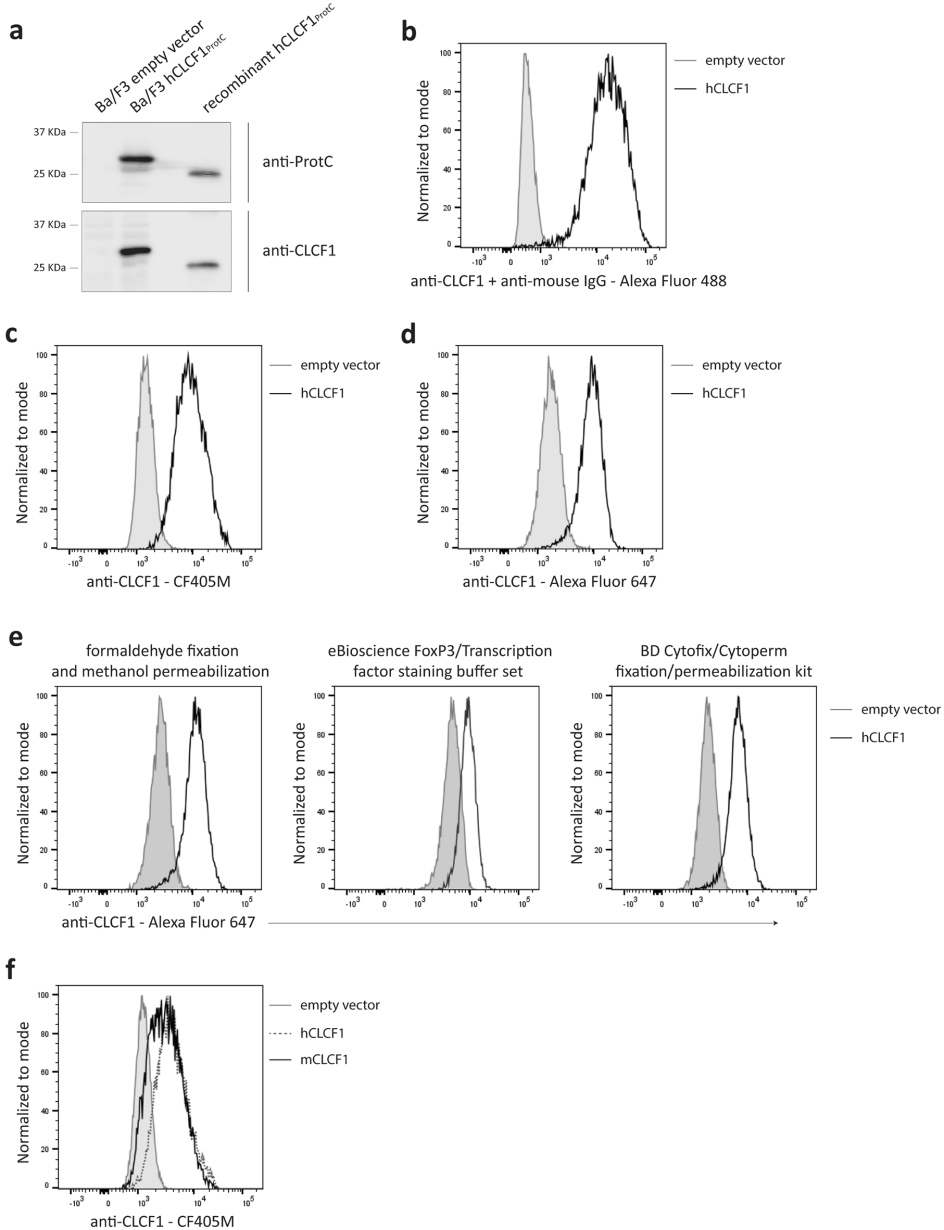
CLCF1 can be detected by flow cytometry in Ba/F3 cells transduced with human and mouse CLCF1 cDNA. Ba/F3 cells were transduced with empty pMX retroviruses or pMX coding for protein C epitope-tagged human or mouse CLCF1 cDNA. (**a**) hCLCF1 expression was confirmed by Western Blot using mAbs specific for the tag (anti-ProtC) or for CLCF1 (anti-CLCF1). Ba/F3 cells transduced with empty pMX retroviruses (Ba/F3 empty vector) and recombinant hCLCF1 produced in *E. coli* (10 ng) were used as controls. The molecular weight difference between the recombinant hCLCF1 and the hCLCF1 from Ba/F3 cells is seemingly due to post-transcriptional modifications in eukaryote cells. Western Blot images were cropped. Original blots are presented in Fig. S4. (n = 5) (**b**–**d**) Cells were fixed and permeabilized with formaldehyde and methanol. Flow cytometry fluorescence signal in control (filled grey histograms) or hCLCF1 (black line histograms) expressing Ba/F3 cells was obtained using (**b**) indirect detection with an anti-CLCF1 mAb followed by an Alexa Fluor 488-conjugated secondary antibody (n = 4) or (**c**,**d**) direct detection with an anti-CLCF1 mAb conjugated to (**c**) CF405M (n = 7) or (**d**) Alexa Fluor 647 (n = 5). Control stainings with secondary antibodies and/or unstained cells are presented in Fig. S3a–c. (**e**) Cells were fixed and permeabilized with formaldehyde and methanol (left panel), the eBioscience FoxP3/Transcription factor staining buffer set (middle panel) or the BD Cytofix/Cytoperm fixation/permeabilization kit (right panel). Flow cytometry fluorescence signal was detected in control (filled grey histogram) or hCLCF1 expressing (black line histogram) Ba/F3 cells stained with an Alexa Fluor 647-conjugated anti-CLCF1 mAb. (n = 3) (**f**) Cells were fixed and permeabilized with formaldehyde and methanol. Flow cytometry fluorescence signal was detected in control (filled grey histogram), human (dotted line histogram) or mouse (solid line histogram) CLCF1 expressing Ba/F3 cells that were stained with a CF405M-conjugated anti-CLCF1 mAb. (n = 3).

In order to identify a suitable antibody to detect CLCF1 protein expression by flow cytometry, we first generated a derivative of the mouse pro-B cell line Ba/F3 expressing a protein C-tagged human CLCF1 construct (Ba/F3 hCLCF1_ProtC_). CLCF1 expression was confirmed by Western Blot (WB) using an anti-protein C tag mAb and an anti-CLCF1 mAb (Clone #138815) (Fig. 1a). After confirming CLCF1 expression by WB, we tested whether the anti-CLCF1 mAb could also be used to detect CLCF1 by flow cytometry on fixed and permeabilized Ba/F3 hCLCF1_ProtC_ cells. To do so, we assessed both indirect (i.e. using unlabelled anti-CLCF1 mAb with a fluorochrome-conjugated anti-mouse IgG; Fig. 1b) and direct detection with CF405M-conjugated (Fig. 1c) or Alexa Fluor 647-conjugated anti-CLCF1 mAb (Fig. 1d). Our results showed that CLCF1 is quantifiable by flow cytometry in permeabilized Ba/F3 hCLCF1_ProtC_ cells using both indirect and direct detection. We also demonstrated that the conjugated anti-CLCF1 mAb is compatible with two commercial fixation/permeabilization kits (eBioscience FoxP3/Transcription factor staining buffer set and BD Cytofix/Cytoperm fixation/permeabilization kit), as well as formaldehyde fixation/methanol permeabilization (Fig. 1e).

### The anti-CLCF1 mAb can cross-react with mouse CLCF1

Human and mouse CLCF1 amino acid sequences are highly conserved with 97% identity^7^. We therefore investigated whether the anti-CLCF1 mAb, which is directed against human CLCF1, could cross-react with mouse CLCF1. While the fluorescence signal observed was slightly lower for mouse CLCF1, we showed that both human and mouse CLCF1 could be detected by flow cytometry in transduced Ba/F3 cells (Fig. 1f). This result indicates that the anti-CLCF1 mAb can cross-react with mouse CLCF1, which makes it a useful tool for the study of CLCF1 expression and function in both human and mouse tissues.

### The anti-CLCF1 mAb interferes with the CLCF1-CRLF1 interaction

**Figure 2:**
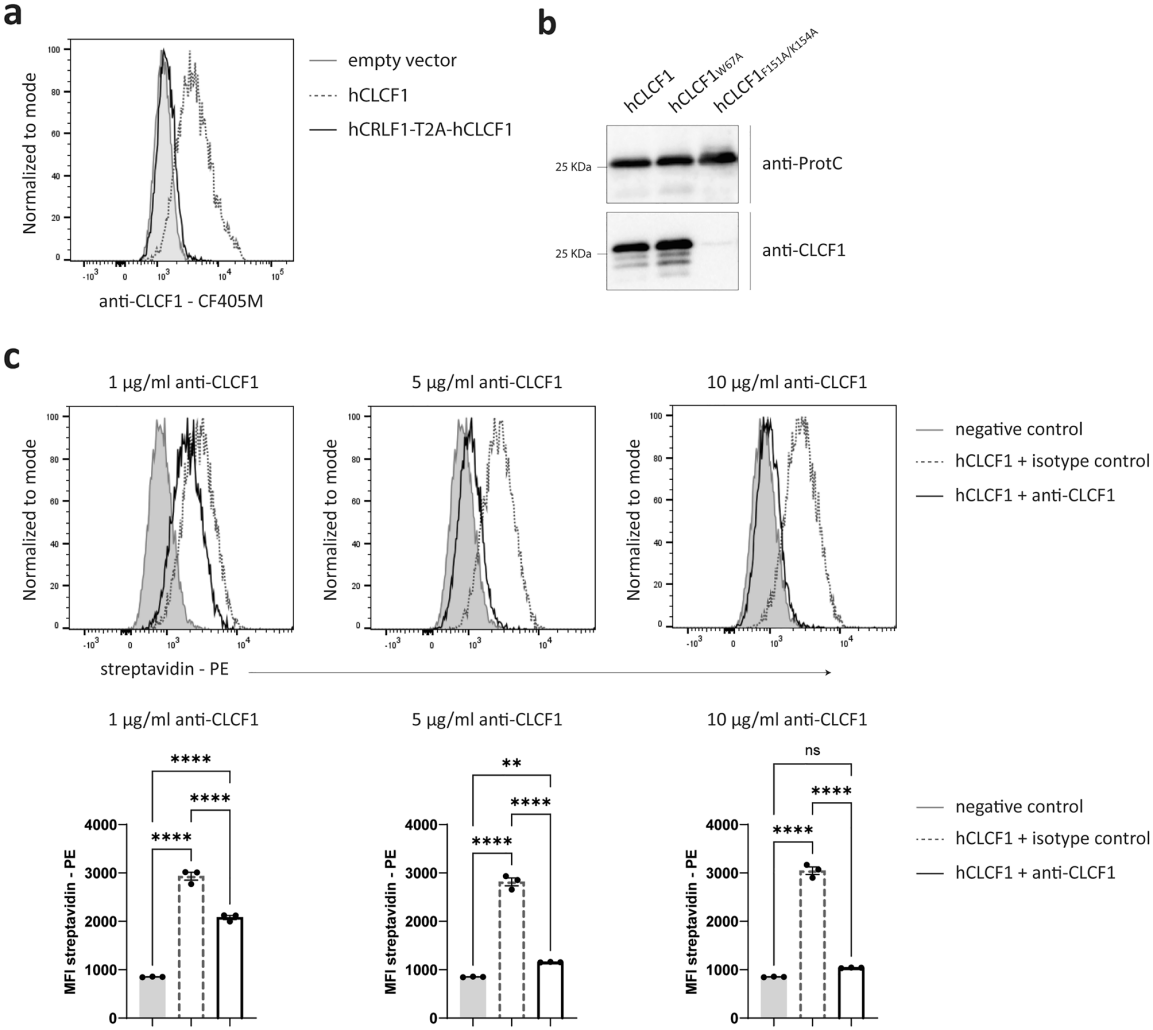
The anti-CLCF1 mAb recognizes an epitope that encompasses CLCF1 binding site III and can inhibit CLCF1-CRLF1 interaction. (**a**) Ba/F3 cells were transduced with empty pMX retroviruses (empty vector; filled grey histogram) or pMX coding for human CLCF1 (hCLCF1; dotted line histogram) or human CLCF1 and CRLF1 (hCRLF1-T2A-hCLCF1; solid line histogram). CRLF1-induced CLCF1 secretion was inhibited by adding brefeldin A for 4 h and cells were fixed and permeabilized with formaldehyde and methanol. Flow cytometry fluorescence signal was detected with a CF405M-conjugated anti-CLCF1 mAb. (n = 4) (**b**) Recombinant wild-type CLCF1 (hCLCF1), binding site I mutant CLCF1 (hCLCF1_W67A_) or binding site III mutant CLCF1 (hCLCF1_F151A/K154A_) were produced in *E. coli* and subjected to Western Blot analysis using anti-ProtC (upper panel) or anti-CLCF1 (lower panel) mAbs. Western Blot images were cropped. Original blots are presented in Fig. S4. (n = 3) (**c**) Ba/F3 cells were transduced with a pMX derivative coding for a hCRLF1-sggg linker-GPI anchor fusion protein and incubated with biotinylated hCLCF1 in the presence of anti-CLCF1 mAb (solid line histogram) or isotype control (dotted line histogram). Binding of CLCF1 to cell surface-bound CRLF1 was detected using PE-conjugated streptavidin. Fluorescence signal of PE-conjugated streptavidin background binding in the absence of hCLCF1 is used as a negative control (filled grey histogram). Bar graphs represent mean fluorescence intensity (MFI) of PE-conjugated streptavidin ± SEM. Statistical significance was assessed using an ANOVA with a Bonferroni post-hoc test, with **p < 0.01 and ****p < 0.0001. (n = 6).

In order to be secreted, CLCF1 needs to form a composite cytokine complex with CRLF1^4^. We investigated whether the presence of CRLF1 could interfere with the detection of CLCF1 by flow cytometry. For this purpose, Ba/F3 cells were transduced with a CLCF1 cDNA or a bi-cistronic cDNA coding for both CRLF1 and CLCF1 (CRLF1-T2A-CLCF1). CRLF1 and CLCF1 expression was confirmed in transduced Ba/F3 cells by Taqman real-time quantitative PCR (RT-qPCR) (data not shown). Interestingly, despite blocking CRLF1-induced secretion of CLCF1 with brefeldin A, CLCF1 was undetectable in cells co-expressing CLCF1 and CRLF1 (Fig. 2a). This suggests that CRLF1 masks the epitope recognized by the anti-CLCF1 mAb, thus preventing the detection of CLCF1 in the CLCF1/CRLF1 complex (Fig. 2a).

Like IL-6 and CNTF, CLCF1 has three receptor binding sites^17^. To investigate the interaction interface between CLCF1 and the anti-CLCF1 mAb, we produced recombinant CLCF1 with site-specific mutations that inactivate CLCF1 binding site I (W67A) or site III (F151A/K154A)^17^. Previous studies have indicated that CLCF1 site I binds CNTFRα, while site III binds LIFRβ^17^. Mutation of CLCF1 binding site III also prevents CRLF1-mediated CLCF1 secretion^17^. We observed by WB that, while the mutation of CLCF1 binding site I has no effect, the anti-CLCF1 mAb is unable to detect the CLCF1 site III mutant (Fig. 2b). This data suggests that the epitope recognized by the anti-CLCF1 mAb encompasses the site III of CLCF1.

To further test whether the anti-CLCF1 mAb and CRLF1 both bind CLCF1 site III, we investigated the effect of the anti-CLCF1 mAb on the interaction between CLCF1 and CRLF1. To do so, we used Ba/F3 cells that were transduced with a retrovirus coding for a hCRLF1-sggg linker-GPI anchor chimeric fusion protein (i.e. a membrane-bound derivative of CRLF1). Using these cells, the binding of biotinylated CLCF1 to membrane-bound CRLF1 can be detected by flow cytometry using PE-conjugated streptavidin. Increasing concentrations of anti-CLCF1 mAb were added to determine whether the anti-CLCF1 mAb could inhibit the binding of CLCF1 to CRLF1. Our results showed a dose-dependent blocking of CLCF1 binding to CRLF1 in the presence of the anti-CLCF1 mAb. Indeed, we observed a partial block in the presence of 1 μg/ml of anti-CLCF1 mAb (0.67 molar ratio between anti-CLCF1 mAb and CLCF1) or 5 μg/ml (3.3 molar ratio), and a near complete block in the presence of 10 μg/ml (6.7 molar ratio) (Fig. 2c). This data shows that the anti-CLCF1 mAb can prevent CLCF1-CRLF1 interaction, likely by competing with CRLF1 for binding on CLCF1 site III.

### The anti-CLCF1 mAb can inhibit CLCF1 biological activity in vitro

**Figure 3:**
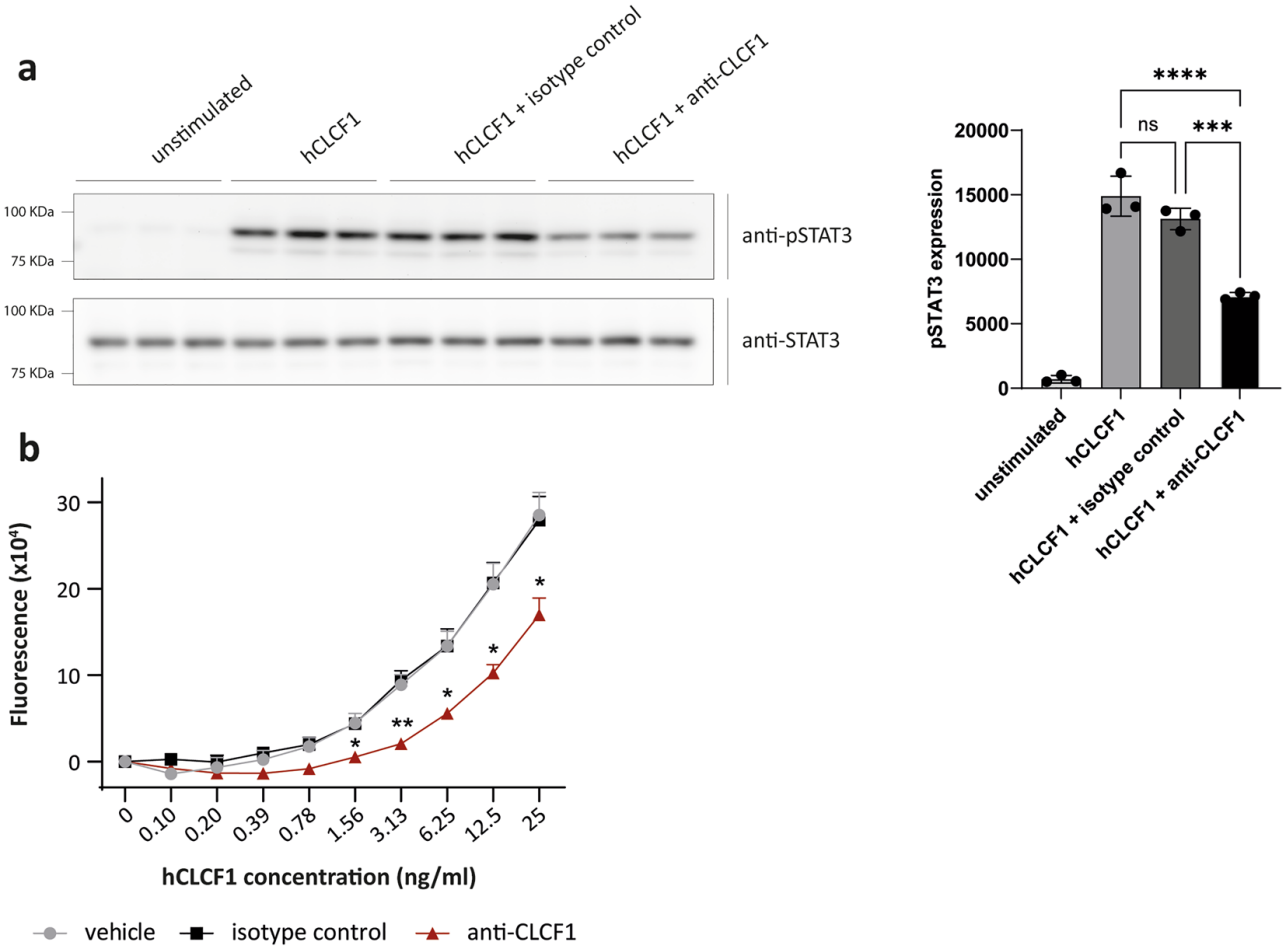
The anti-CLCF1 mAb can inhibit CLCF1 biological activity in vitro*.* Ba/F3 cells were transduced with pMX retroviruses coding for the three receptor chains (CNTFRα, LIFRβ and gp130) of the CNTF receptor (Ba/F3 CNTFR). (**a**) Ba/F3 CNTFR triplicate cell cultures were stimulated with hCLCF1 (25 ng/ml) in the presence of vehicle, isotype control or anti-CLCF1 mAb (2.5μg/ml). Cell lysates were subjected to Western Blot analysis with mAbs specific for phosphorylated (upper panel) or total STAT3 (lower panel). Western Blot signal intensity was quantified using the ImageJ software. Western Blot images were cropped. Original blots are presented in Fig. S4. Bar graph represents pSTAT3 signal intensity ± SEM. Statistical significance was assessed using an ANOVA with a Bonferroni post-hoc test, with ***p < 0.001 and ****p < 0.0001. (n = 3) (**b**) Proliferation of Ba/F3 CNTFR cells in response to increasing concentrations of hCLCF1 was assessed in the presence of 2.5 μg/ml of anti-CLCF1 mAb (red triangles), isotype control (black squares) or vehicle (grey circles). Ba/F3 proliferation was measured in triplicates using an alamarBlue fluorometric assay. Statistical significance was assessed using a one-way ANOVA and a Tukey post-hoc test for each concentration point of hCLCF1, with *p < 0.05 and **p < 0.01. (n = 5).

In addition to its role in the CLCF1-CRLF1 interaction, CLCF1 binding site III is also involved in the recruitment and activation of the LIFRβ receptor chain of the tripartite CNTFR^17^. Since the anti-CLCF1 mAb seems to block CLCF1 site III, we hypothesized that the anti-CLCF1 mAb could inhibit CLCF1 biological activity on its receptor by blocking the interaction with the receptor signaling chain LIFRβ. To test this, we used Ba/F3 cells that were transduced with cDNAs coding for the 3 subunits of the CNTFR (i.e. CNTFRα/LIFRβ/gp130). In these cells, activation of the CNTFR with cytokines leads to the activation of the JAK/STAT3 signaling pathway, which induces cell proliferation. Using these CNTFR-expressing Ba/F3 cells, we demonstrated that 2.5 μg/ml of anti-CLCF1 mAb (3.3 molar excess) is sufficient to produce a significant reduction in STAT3 phosphorylation (Fig. 3a), and that this decrease in CNTFR activation is associated with a matching reduction in CLCF1-induced proliferation (Fig. 3b). Altogether, these results show that the anti-CLCF1 mAb can inhibit CLCF1 activity in vitro.

### Detection of CLCF1 in mouse T cells

We next sought to utilize our CLCF1 detection methodology to investigate CLCF1 expression in mouse splenocytes. To assess the specificity of the flow cytometry fluorescence signal, we used splenocytes from *Clcf1* conditional knock-out mice. This mouse model was obtained by breeding *Clcf1* flox/flox mice (i.e. mice that had LoxP sites inserted either side of the exon 3 in the *Clcf1* gene using CRISPR-Cas9 technology^18^) with Vav-iCre transgenic mice to generate offspring with a constitutive knock-out of *Clcf1* in immune cells. Taqman RT-qPCR was used to confirm the Vav-iCre induced deletion of *Clcf1* in our KO splenocytes (Fig. S1).

**Figure 4:**
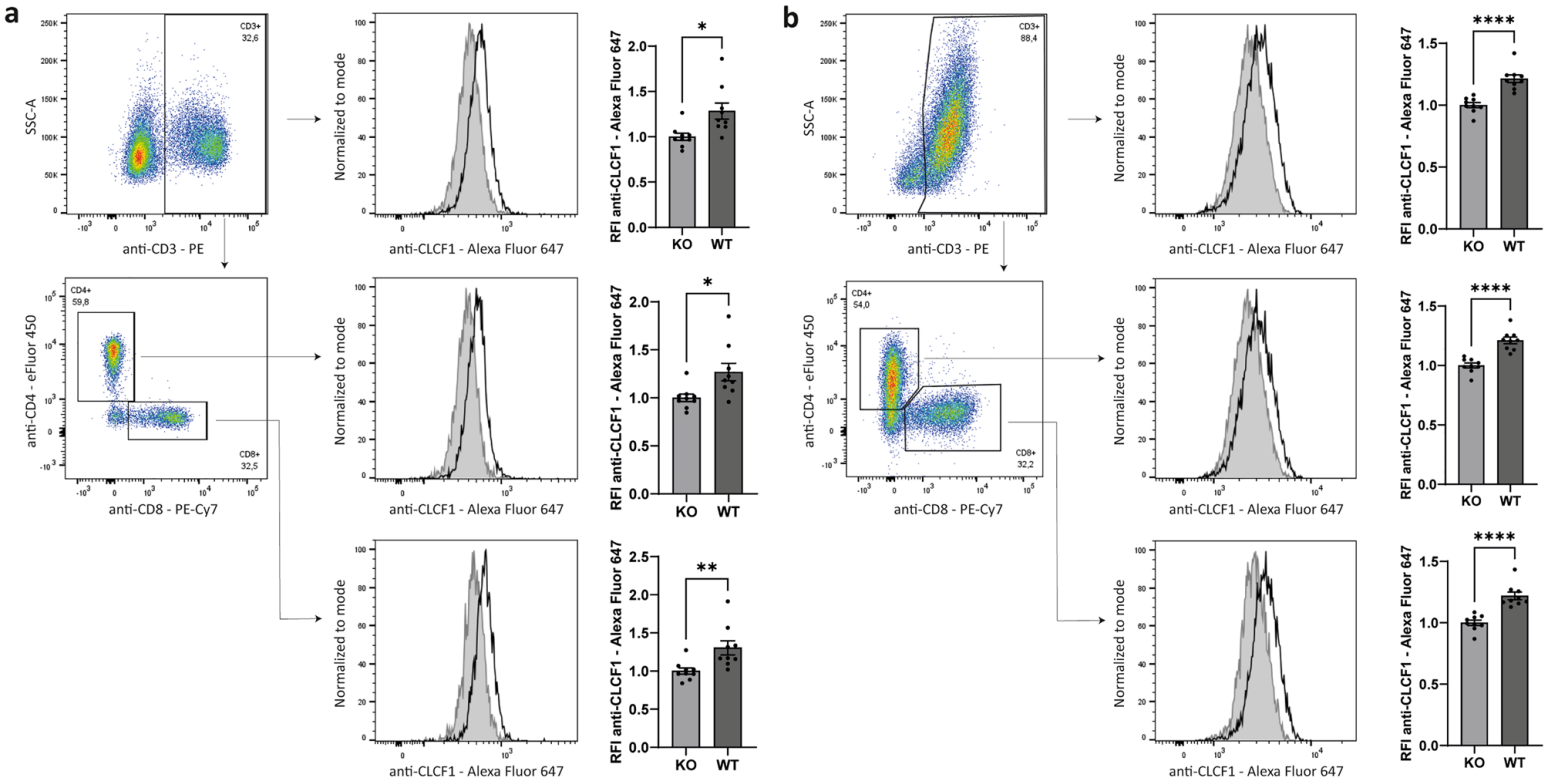
CLCF1 is expressed by mouse CD4^+^ and CD8^+^ T cells. Mouse splenocytes were analysed (**a**) upon isolation or (**b**) after 3 d of activation with plate-bound anti-TCR and soluble anti-CD28. (**a**,**b**) All cells were stimulated with PMA/ionomycin/brefeldin A for 4 h, and fixed and permeabilized with the eBioscience FoxP3/Transcription factor staining buffer set. The panels on the left show the immune population investigated (top : CD3^+^; middle and bottom : CD4^+^ and CD8^+^ gated on CD3^+^ cells). The panels to the right compare the fluorescence signal of the Alexa fluor 647-conjugated anti-CLCF1 mAb of cells isolated from *Clcf1* knock-out mice (KO; filled grey histogram) or wild-type mice (WT; black line histogram). The bar graphs show the relative fluorescence intensity (RFI) for the Alexa Fluor 647-conjugated anti-CLCF1 mAb ± SEM. Statistical significance was assessed using Student’s t test, with *p < 0.05, **p < 0.01, and ****p < 0,0001. Control unstained cells are presented in Fig. S3d,e. Experiments were performed in triplicate, and replicated in three separate experiments.

Using our flow cytometry CLCF1 detection protocol with the *Clcf1* KO cells as control, we observed CLCF1 expression in total T cells (Fig. 4a upper panel), as well as in CD4^+^ and CD8^+^ T cells (Fig. 4a middle and lower panels). We also detected CLCF1 expression in B cells, myeloid cells, and NK cells (Fig. S2). To investigate whether CLCF1 expression in T cells is modulated by activation, we stimulated splenocytes with plate-bound anti-TCR and soluble anti-CD28 for 3 d. The expression pattern of CLCF1 was similar in naïve and activated cells, with expression in both CD4^+^ and CD8^+^ T cells (Fig. 4b).

As CRLF1 promotes CLCF1 secretion^4^ and interferes with CLCF1 detection by the anti-CLCF1 mAb (Fig. 2c), we also assessed CRLF1 mRNA levels by RT-qPCR in unfractionated splenocytes, as well as resting and activated CD4^+^ T cells from wild-type mice. Our results show that CRLF1 expression was minimal/undetectable in all tested conditions with cycle threshold (Ct) values over 37 (data not shown).

### Detection of CLCF1 in mouse Type 1 helper T cells

**Figure 5:**
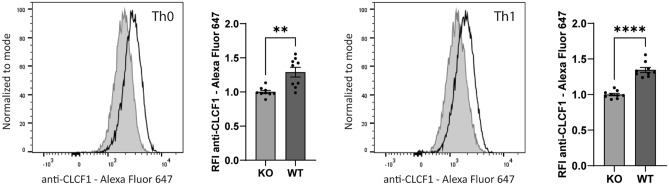
CLCF1 is expressed by differentiated Th1 cells. Mouse splenic CD4^+^ T cells were activated with plate-bound anti-TCR and soluble anti-CD28 alone (Th0) or supplemented with neutralizing anti-IL-4 mAb and mouse IL-12 (10 ng/ml) (Th1) for 5 d. Cells were re-stimulated with PMA/ionomycin/brefeldin A for 4 h, and fixed and permeabilized with the eBioscience FoxP3/Transcription factor staining buffer set. A flow cytometry histogram graph and a bar graph are shown for each stimulation condition (Left : Th0; Right : Th1). The flow cytometry histogram graph compares the fluorescence signal of the Alexa Fluor 647-conjugated anti-CLCF1 mAb of cells isolated from a *Clcf1* KO (grey filled histogram) or WT (black line histogram) mouse. The bar graph represents relative fluorescence intensity (RFI) of the Alexa Fluor 647-conjugated anti-CLCF1 mAb ± SEM. Statistical significance was assessed using Student’s t test, with **p < 0.01 and ****p < 0.0001. Control unstained cells are presented in Fig. S3f. The experiment was performed in triplicate, and replicated in three separate experiments.

The expression levels of many cytokines are altered during Th cell differentiation. CLCF1 is no exception as CLCF1 mRNA is over-expressed in human memory Th17 cells expanded in vitro^14^. To investigate whether our methodology could detect CLCF1 expression in Th cell subtypes, we cultured mouse T cells under non-polarizing conditions (Th0) or conditions favoring Th1 polarization. To induce Th1 polarization, we stimulated CD4^+^ T cells from WT or *Clcf1* conditional KO mice with anti-TCR and anti-CD28 in the presence of anti-IL-4 and IL-12 for 5 d. By comparing the fluorescence signal between the WT and KO cells, we were able to detect CLCF1 expression in cells differentiated under non-polarizing (Th0) and Th1 polarizing conditions (Fig. 5). These results indicate that CLCF1 can be detected in CD4^+^ effector T cells, and validates the use of anti-CLCF1 mAb as a tool to study CLCF1 expression patterns in T cell subpopulations.

## Discussion

Our aim was to develop a mAb-based detection protocol for the cytokine CLCF1 that could easily be combined with other mAbs in multi-color panel flow cytometry. It represents a substitute for our previous approach, which used rabbit polyclonal anti-CLCF1 serum to show CLCF1 expression in a subpopulation of GR-1^+^ cells in the mouse bone marrow^16^. With this current methodology, we managed to detect CLCF1 expression in both unstimulated and activated mouse splenic CD4^+^ and CD8^+^ T cells, as well as in in vitro differentiated Th1 cells. Our approach was validated using transduced mouse Ba/F3 cells overexpressing CLCF1, and primary immune cells deficient for *Clcf1*. RT-qPCR results on splenocytes from *Clcf1* KO mice indicated that CLCF1 mRNA is undetectable in these cells, thus confirming the effectiveness of the *Clcf1* deletion in hematopoietic cells by the Vav-iCre promoter. As the complete KO of *Clcf1* in mice is lethal at P1^19^, this new mouse model will be useful for the future study of CLCF1 immune functions and pathological roles.

CLCF1 was first cloned from activated Jurkat human T cell lymphoma cells^2^. This is consistent with our current observations of CLCF1 expression in resting and activated mouse T cells. Our investigation further showed that this cytokine is expressed in Th1 cells. We believe our methodology could be used in the future to further characterize CLCF1 expression patterns at various stages of T cell activation, as well as in other types of T helper cell differentiation.

Conversely, CLCF1 secretion partner CRLF1 does not seem to be expressed by T cells, as CRLF1 mRNA levels were at the detection limit of RT-qPCR in both splenocytes and CD4^+^ T cells. Therefore, CLCF1 produced by T cells is unlikely to be secreted via the CLCF1-CRLF1 complex. It remains to be investigated whether CLCF1 in T cells could be secreted in complex with an alternative secretion partner^20^, or released via an unconventional pathway as shown for IL-35^21^, which would allow this cytokine to mediate its known immuno-modulating activities on B cells^1,2^ or myeloid cells^8^. Additionally, intracellular CLCF1 in T cells might possess yet to be identified intracrine roles in the control of Th cell differentiation.

Moreover, we showed using CLCF1 mutants that the epitope recognized by the anti-CLCF1 mAb overlaps with CLCF1 binding site III. This site is responsible for the interaction of CLCF1 with either CRLF1 for secretion or LIFRβ for receptor activation^17^. We showed that the anti-CLCF1 mAb cannot detect CLCF1 when its binding site III is masked by CRLF1 in the CLCF1/CRLF1 complex. As CLCF1 could be detected in T cells by flow cytometry, this observation further suggests that the cytokine is not complexed with CRLF1 in these cells. Furthermore, the anti-CLCF1 mAb can prevent the formation of the CLCF1/CRLF1 complex, as well as inhibit the activation of CNTFR, presumably by blocking the recruitment of LIFRβ. CLCF1 is currently being investigated as a potential target for the treatment of various types of cancers, such as gliomas^22^ and non-small cell lung cancer^13^. The group of Dr. Sweet-Cordero is also developing an engineered high-affinity soluble form of CNTFRα (eCNTFR-Fc) that has been shown to sequester CLCF1, therefore inhibiting its oncogenic effects in a lung adenocarcinoma mouse model^23^. Our results suggest that the anti-CLCF1 mAb, which recognizes CLCF1 site III, could potentially be used as an alternative for the neutralization of CLCF1 oncogenic activity in these preclinical models. Moreover, some pathologies, such as focal segmental glomerulosclerosis^24^, might implicate CLCF1 rather than the CLCF1/CRLF1 complex. The anti-CLCF1 mAb inability to bind CLCF1 when in complex with CRLF1 therefore makes it a candidate for the preclinical investigation of selective inhibition of free CLCF1 in these pathologies.

In conclusion, the methodology outlined in this study allows the investigation of normal and pathological CLCF1 expression patterns at a protein level, which will facilitate the study of CLCF1 function in normal or pathological settings, and potentially reveal new roles for this understudied cytokine.

## Methods

### Experimental animals

The *Clcf1* floxed C57BL/6 mouse model with LoxP sites inserted in 5’ and 3’ of the *Clcf1* third exon^18^ was crossbred to wild-type C57BL/6 mice (Strain #000664) for 6 generations. *Clcf1* flox/flox mice were then bred to C57BL/6 Vav-iCre transgenic mice (Strain #008610)^25^ purchased from The Jackson Laboratory (Bar Harbor, ME) to produce experimental animals that were used at 8–12 weeks of age. Age and sex-matched wild-type C57BL/6 mice were used as controls. All procedures were performed in accordance with the Canadian Council on Animal Care guidelines and approved by the Comité de déontologie de l’expérimentation sur les animaux (CDEA), Université de Montréal, and the Comité de protection des animaux du CIUSS de l'Est-de-l’Île-de-Montréal (CPA-CEMTL). All authors complied with the ARRIVE guidelines.

### Generation of transgenic Ba/F3 cell lines

Codon optimized synthetic cDNAs coding for human CLCF1 tagged with the protein C epitope (EDQVDPRLIDGK), human CRLF1-T2A-CLCF1^26^, mouse CLCF1, human CRLF1-SGGG linker-GPI anchor (CNTFR a.a 346-372) fusion protein, and human CNTFRα were generated by GeneArt (Thermo Fisher Scientific, Burlington ON). Human LIFRβ and human gp130 cDNAs were obtained from Sino Biologicals (Wayne, PA). All cDNAs were subcloned into the pMX retroviral expression vector^27^. VSV-G pseudo-typed recombinant retroviruses were transduced in the IL-3-dependent murine pro-B cell line Ba/F3 (Creative Bioarray, Shirley NY) in the presence of polybrene (8 μg/ml). Stable transgenic Ba/F3 derivatives were selected with puromycin (1 μg/ml). Ba/F3 cells expressing the tripartite CNTF receptor (CNTFRα/LIFRβ/gp130; "Ba/F3 CNTFR") were positively selected using in house-produced recombinant human CLCF1 (50 ng/ml). For regular subculturing, Ba/F3 cells were expanded with recombinant murine IL-3 (10 ng/ml; PeproTech, Cranbury NJ) in RPMI 1640 medium supplemented with fetal bovine serum (FBS; 10%), L-glutamine (4 mM), penicillin (100 U/ml), streptomycin (100 μg/ml), HEPES pH 7.4 (10 mM) and β-mercaptoethanol (0.05 mM) ("complete RPMI medium").

### Detection of CLCF1 by Western Blot

Transduced Ba/F3 cells were lysed in SDS-PAGE sample loading buffer. Recombinant human protein C-tagged wild-type or mutant^17^ CLCF1 proteins were produced in *E. coli* BL21 (DE3; Thermo Fisher Scientific) as previously described^28^. Cell lysates and proteins were subjected to 12% polyacrylamide SDS-PAGE, and electrotransferred to polyvinylidene difluoride (PVDF) membranes. Membranes were probed with anti-CLCF1 (Clone #138815; R&D systems, Minneapolis MN) or anti-protein C (HPC4; GenScript, Piscataway NJ) mAbs, followed by horseradish peroxidase (HRP)-conjugated anti-mouse and anti-rabbit IgG respectively. Chemiluminescence signal was detected using an ImageQuant LAS 4000 (GE Healthcare, Chicago IL), and processed using the ImageJ software.

### Detection of CLCF1 in Ba/F3 cells by intracellular flow cytometry

CRLF1 and CLCF1 co-expressing Ba/F3 cells were treated with brefeldin A (3 μg/ml) in complete RPMI medium for 4 h. All cells were stained with eFluor 506 Fixable Viability Dye (Thermo Fisher Scientific) on ice for 15 min, fixed in 2% formaldehyde at room temperature for 10 min and permeabilized with 100% cold methanol on ice for 10 min. When indicated, Ba/F3 cells were alternatively fixed and permeabilized using either the eBioscience FoxP3/Transcription factor staining buffer set (Thermo Fisher Scientific) or the BD Cytofix/Cytoperm fixation/permeabilization kit (BD Biosciences, Franklin Lakes NJ) according to manufacturer’s instructions. CLCF1 was indirectly detected with anti-CLCF1 mAb (1 μg/ml; Clone #138815; R&D systems) and Alexa Fluor 488-conjugated anti-mouse IgG (Thermo Fisher Scientific). For direct detection of CLCF1, CF405M-conjugated anti-CLCF1 mAb (3 μg/ml) or Alexa Fluor 647-conjugated anti-CLCF1 mAb (1 μg/ml) were used. Both mAb conjugations were done according to manufacturer’s instructions using either the CF405M Mix-n-Stain (Sigma-Aldrich, Oakville ON) or the Alexa Fluor 647 (Thermo Fisher Scientific) antibody labeling kits. Fluorescence was quantified using a FacsCanto II or FACSymphony A1 flow cytometer (BD Biosciences). Data were analyzed using the FlowJo software (BD Biosciences).

### Binding assay

Recombinant biotinylated human CLCF1^28^ (0.5 μg/ml), and anti-CLCF1 mAb (1, 5 or 10 μg/ml) or matching concentrations of mouse IgG2b isotype control (clone #20116; R&D systems) were pre-incubated together for 1 h at 37 °C. Ba/F3 transduced derivatives were incubated with the anti-CLCF1 mAb-CLCF1 mixes in PBS containing 0.1% BSA on ice for 1 h. Cell membrane-bound CLCF1 was detected with PE-conjugated streptavidin (Thermo Fisher Scientific). Non-viable cells were excluded using eFluor 506 Fixable Viability Dye. Fluorescence was assessed using a FACSymphony A1 flow cytometer. Data were analyzed using the FlowJo software.

### STAT3 tyrosine phosphorylation assay

Recombinant human CLCF1 (25 ng/ml), and anti-CLCF1 mAb or mouse IgG2b isotype control (2.5 μg/ml) were pre-incubated together for 1 h at 37˚C. Ba/F3 CNTFR cells (1 × 10^6^ cells/condition) were serum-starved for 4 h and then stimulated with the anti-CLCF1 mAb-CLCF1 mixes in PBS containing 0.1% BSA for 30 min at 37˚C. Cells were lysed in 50 mM Tris pH 7.5, 150 mM NaCl, 1% Nonidet P40, 0.5% sodium deoxycholate, 0.1% SDS, 1 × cOmplete™ protease inhibitors cocktail (Roche, Sigma-Aldrich, Oakville, ON) and 1 × Halt phosphatase inhibitors cocktail (Thermo Fisher Scientific). Proteins were subjected to 7.5% polyacrylamide SDS-PAGE and electrotransferred to PVDF membranes. Membranes were probed with anti-STAT3 or anti-pSTAT3 (Tyr705) mAbs (Cell Signaling Technology, Danvers MA), followed by HRP-conjugated anti-rabbit IgG. Chemiluminescence signal was detected using an ImageQuant LAS 4000 (GE Healthcare), and processed using the ImageJ software.

### Proliferation assay

Recombinant human CLCF1 (0.1–25 ng/ml), and anti-CLCF1 mAb or mouse IgG2b isotype control (2.5 μg/ml) were pre-incubated together for 1 h at 37˚C. Ba/F3 CNTFR cells (1 × 10^4^ cells/well in 96-well plates) were incubated in triplicates with the anti-CLCF1 mAb-CLCF1 mixes for 72 h in RPMI 1640 supplemented with 5% FBS. Proliferation was assessed using an alamarBlue fluorometric assay (BioRad, Hercules CA). Fluorescence at 590 nm was quantified with a Synergy H1 plate reader (BioTek, Winooski VT).

### Detection of CLCF1 in mouse splenocytes

Mouse mononucleated splenocytes were isolated using histopaque density gradient centrifugation, and cultivated in complete RPMI medium. Stimulated splenocytes were incubated with coated anti-TCR (5 μg/ml) (H57-597; homemade) and soluble anti-CD28 (1 μg/ml; BD Biosciences) for 3 d. Non-stimulated and stimulated cells were incubated with a cell stimulation cocktail plus protein transport inhibitors (Thermo Fisher Scientific) in complete RPMI medium for 4 h. Cells were labelled in PBS on ice for 45 min with eFluor 506 Fixable Viability Dye, BV711-conjugated anti-CD19 (Clone 6D5; BioLegend, San Diego CA), PE-conjugated anti-CD3ε (Clone 145-2C11), eFluor 450-conjugated anti-CD4 (Clone RM4-5), PE-Cy7-conjugated anti-CD8α (Clone 53–6.7), APC-eFluor 780-conjugated anti-CD11b (Clone M1/70), PerCP-Cyanine5.5-conjugated anti-NK1.1 (Clone PK136) (all from Thermo Fisher Scientific). Surface-labelled cells were fixed and permeabilized using the FoxP3/Transcription factor staining buffer set, incubated with 2% normal rat serum (STEMCELL Technologies, Vancouver BC) at room temperature for 15 min, and stained with Alexa Fluor 647-conjugated anti-CLCF1 mAb (1 μg/ml) at room temperature for 45 min. Fluorescence was quantified using a FACSymphony A1 flow cytometer. Data were analyzed using the FlowJo software.

### Detection of CLCF1 in Th1 differentiated T cells

Mouse mononucleated splenocytes were isolated with histopaque density gradient centrifugation, and CD4^+^ T cells were purified with the EasySep mouse CD4^+^ T cells Isolation kit (STEMCELL Technologies). Cells were stimulated for 5 d in complete RPMI medium with coated anti-TCR (5 μg/ml) and soluble anti-CD28 (1 μg/ml) alone (i.e. Th0), or supplemented with soluble anti-mouse IL-4 (10 μg/ml; clone BVD4-1D11, BD Biosciences) and murine IL-12 (10 ng/ml; PeproTech) (i.e. Th1). Cell culture medium was changed on day 3. On day 5, differentiated CD4^+^ T cells were processed for flow cytometry analysis as described above. Briefly, cells were stained with eFluor 506 Fixable Viability Dye, fixed and permeabilized with the FoxP3/Transcription factor staining buffer set, and stained with Alexa Fluor 488-conjugated anti-IFN-γ (Clone XMG1.2), PerCP-Cyanine5.5-conjugated anti-T-bet (all from Thermo Fisher Scientific) and Alexa Fluor 647-conjugated anti-CLCF1 mAb. Fluorescence was quantified using a FACSymphony A1 flow cytometer. Data were analyzed using the FlowJo software.

### Detection of CLCF1 and CRLF1 mRNA

Mouse unfractionated splenocytes or CD4^+^ T cells were lysed in TRIzol (Thermo Fisher Scientific), chloroform was added, and total RNA was isolated from the aqueous phase using the RNeasy kit (Qiagen, Toronto ON) according to manufacturer’s instructions. TaqMan real-time quantitative RT-PCR analysis was conducted by the Genomics platform at the Institute for Research in Immunology and Cancer (Université de Montréal, Montréal QC).

### Statistical analysis

Unpaired Student's t tests or one-way ANOVAs with a Bonferroni or Tukey post-hoc test were used where appropriate. The results presented in the figures are marked using asterisks with *p < 0.05, **p < 0.01, ***p < 0.001, and ****p < 0.0001. Data were analyzed using the GraphPad prism software (La Jolla CA).

### Supplementary Information

Supplementary Figures.

## Data Availability

All data generated or analysed during this study are included in this published article (and its Supplementary Information files).
