# Supplementary material for: Detection of CLCF1 protein expression by flow cytometry
Source: Sci Rep. 2024 Jun 10;14:13344. doi: 10.1038/s41598-024-64101-9 (PMC11164924; doi:10.1038/s41598-024-64101-9)
Supplement: Supplementary file 1 — Supplementary Figures. [file 41598_2024_64101_MOESM1_ESM.docx]

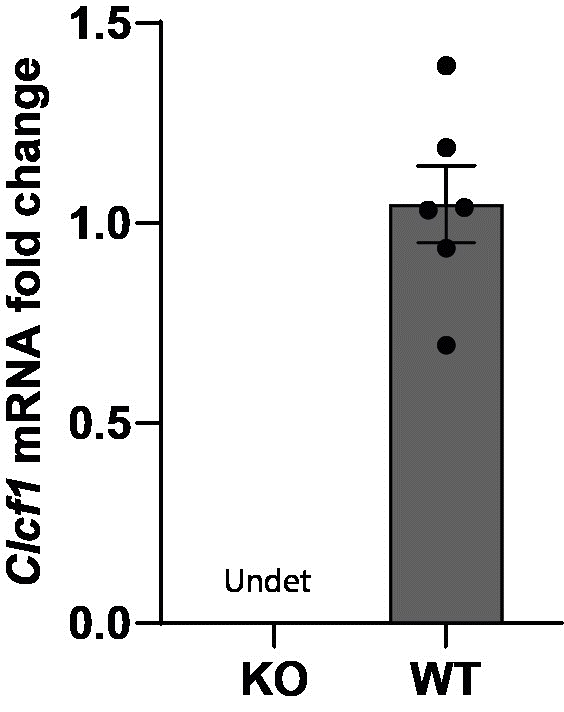


**Supplementary Figure S1. CLCF1 mRNA is undetectable in splenocytes from *Clcf1* knock-out mice.** Splenocytes from wild-type (WT) and *Clcf1* knock-out (KO) mice were stimulated with PMA/ionomycin/brefeldin A for 4 h and processed for Taqman real-time quantitative RT-PCR. Bar graph shows mRNA fold change for *Clcf1* ± SEM. PCR product was undetectable (Undet) in all samples isolated from *Clcf1* knock-out mice.


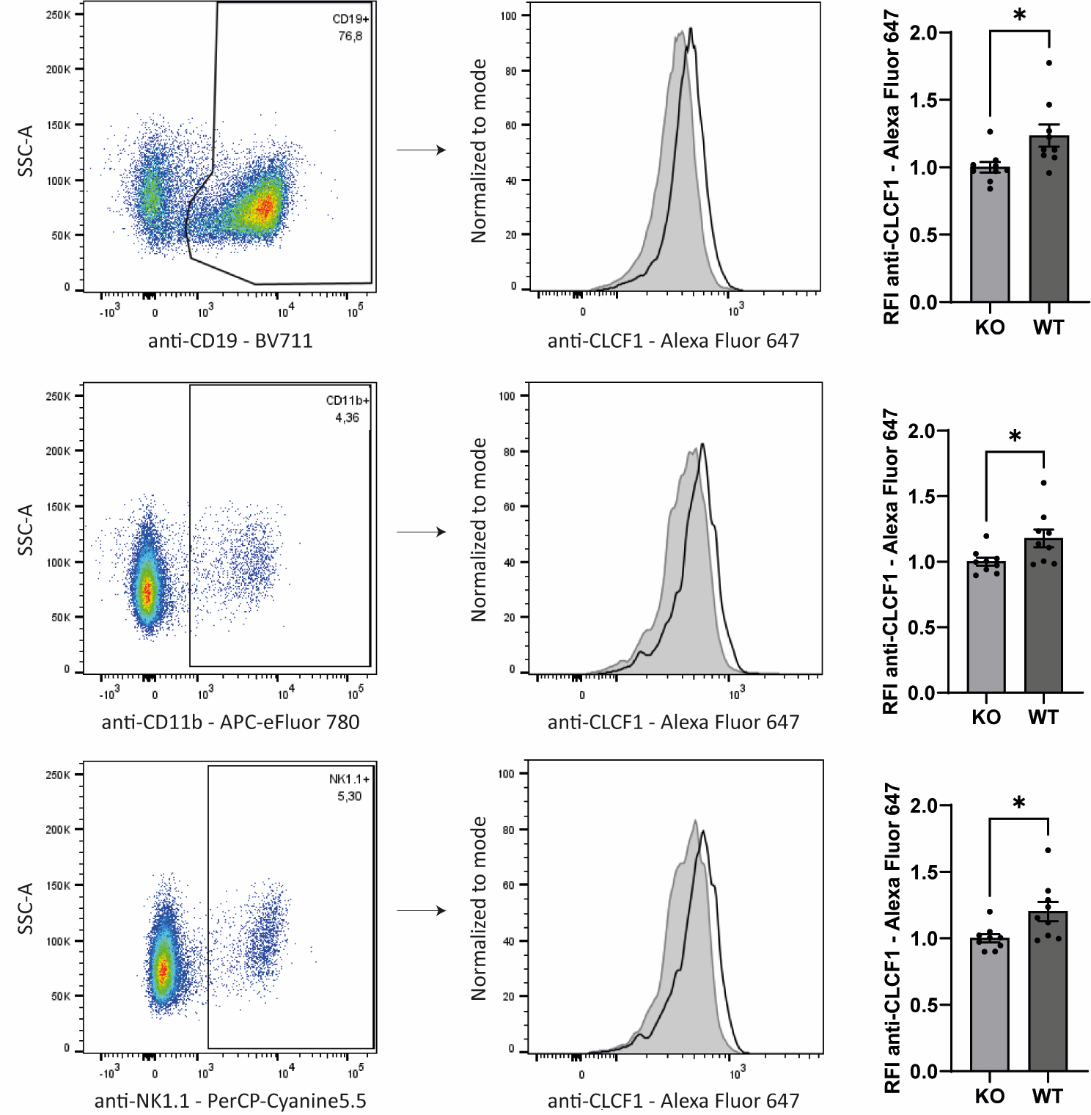


**Supplementary Figure S2. CLCF1 is expressed by B, myeloid and NK cells.** Mouse splenocytes were stimulated with PMA/ionomycin/brefeldin A for 4 h and fixed and permeabilized with the eBioscience FoxP3/Transcription factor staining buffer set. The panels on the left show the immune population investigated (top : B cells, CD19^+^ ; middle : myeloid cells, CD11b^+^ ; bottom : NK cells, NK1.1^+^). The panels to the right compare the fluorescence signal of the Alexa Fluor 647-conjugated anti-CLCF1 mAb of cells isolated from *Clcf1* KO (filled grey histogram) or WT (black line histogram) mice. Histograms were smoothed. The bar graphs show the relative fluorescence intensity (RFI) for the Alexa Fluor 647-conjugated anti-CLCF1 mAb ± SEM. Statistical significance was assessed using Student’s t test, with *p<0.05. (n=3 in triplicates)


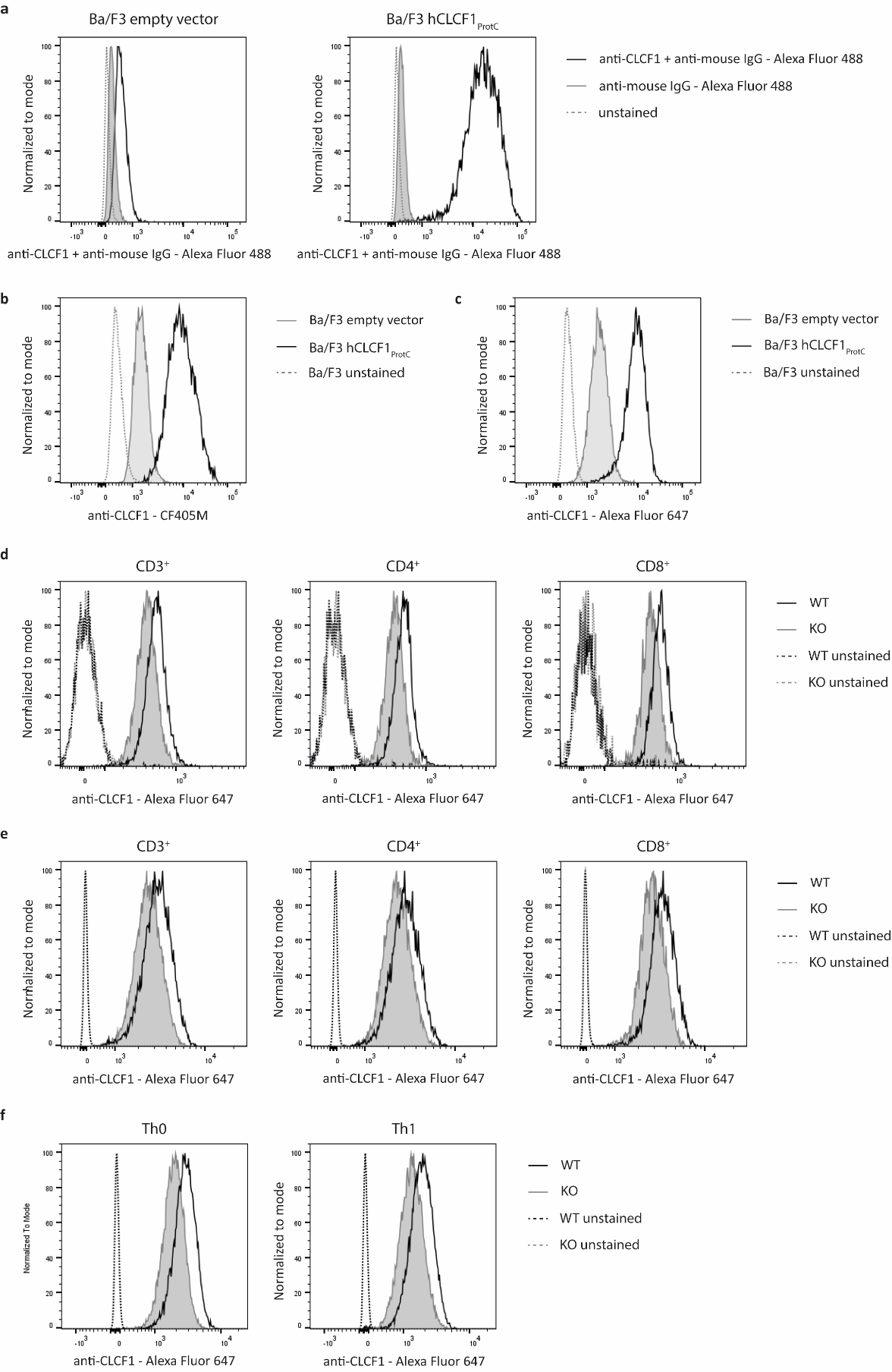


**Supplementary Figure S3. Control stainings for the anti-CLCF1 flow cytometry mAb.** Histograms show control stainings with secondary antibodies and/or unstained cells for (a) Figure 1b, (b) Figure 1c, (c) Figure 1d, (d) Figure 4a, (e) Figure 4b, and (f) Figure 5.


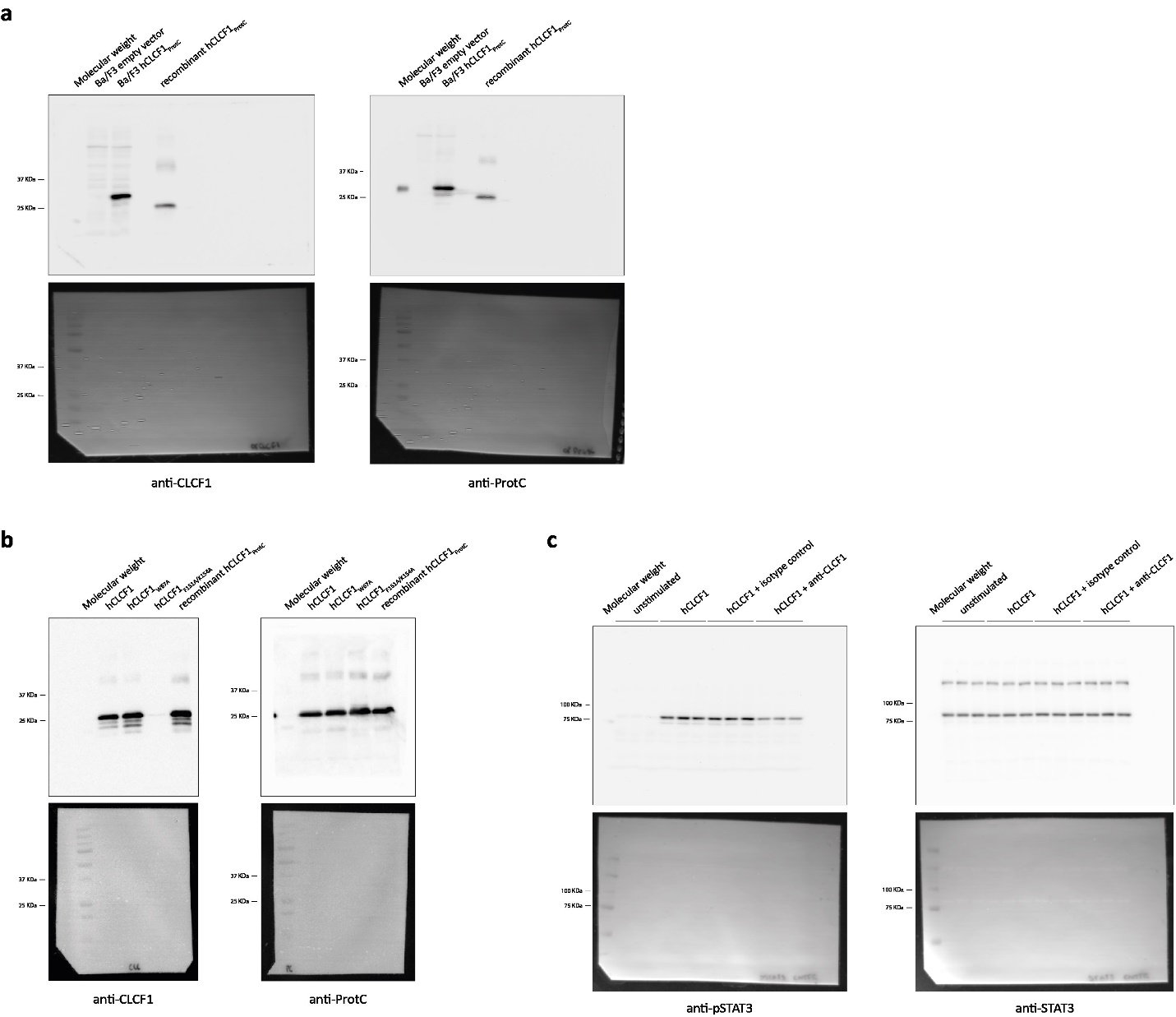


**Supplementary Figure S4. Original Western Blot images.** Original Western Blot images in chemiluminescence (top) and incandescent light (bottom) for (a) Figure 1a, (b) Figure 2b, and (c) Figure 3a.
